# Hybrid solid electrolyte enabled dendrite-free Li anodes for high-performance quasi-solid-state lithium-oxygen batteries

**DOI:** 10.1093/nsr/nwaa150

**Published:** 2020-07-02

**Authors:** Jin Wang, Gang Huang, Jun-Min Yan, Jin-Ling Ma, Tong Liu, Miao-Miao Shi, Yue Yu, Miao-Miao Zhang, Ji-Lin Tang, Xin-Bo Zhang

**Affiliations:** Key Laboratory of Automobile Materials, Ministry of Education, Department of Materials Science and Engineering, Jilin University, Changchun 130022, China; State Key Laboratory of Rare Earth Resource Utilization, Changchun Institute of Applied Chemistry, Chinese Academy of Sciences, Changchun 130022, China; Materials Science and Engineering, Physical Science and Engineering Division, King Abdullah University of Science and Technology, Thuwal 23955-6900, Saudi Arabia; Key Laboratory of Automobile Materials, Ministry of Education, Department of Materials Science and Engineering, Jilin University, Changchun 130022, China; State Key Laboratory of Rare Earth Resource Utilization, Changchun Institute of Applied Chemistry, Chinese Academy of Sciences, Changchun 130022, China; State Key Laboratory of Rare Earth Resource Utilization, Changchun Institute of Applied Chemistry, Chinese Academy of Sciences, Changchun 130022, China; Key Laboratory of Automobile Materials, Ministry of Education, Department of Materials Science and Engineering, Jilin University, Changchun 130022, China; State Key Laboratory of Rare Earth Resource Utilization, Changchun Institute of Applied Chemistry, Chinese Academy of Sciences, Changchun 130022, China; State Key Laboratory of Rare Earth Resource Utilization, Changchun Institute of Applied Chemistry, Chinese Academy of Sciences, Changchun 130022, China; State Key Laboratory of Electroanalytical Chemistry, Changchun Institute of Applied Chemistry, Chinese Academy of Sciences, Changchun 130022, China; State Key Laboratory of Electroanalytical Chemistry, Changchun Institute of Applied Chemistry, Chinese Academy of Sciences, Changchun 130022, China; State Key Laboratory of Rare Earth Resource Utilization, Changchun Institute of Applied Chemistry, Chinese Academy of Sciences, Changchun 130022, China

**Keywords:** lithium-oxygen batteries, dendrite suppression, Li anodes, hybrid solid electrolyte, LAGP, PVDF-HFP

## Abstract

The dendrite growth of Li anodes severely degrades the performance of lithium-oxygen (Li-O_2_) batteries. Recently, hybrid solid electrolyte (HSE) has been regarded as one of the most promising routes to tackle this problem. However, before this is realized, the HSE needs to simultaneously satisfy contradictory requirements of high modulus and even, flexible contact with Li anode, while ensuring uniform Li^+^ distribution. To tackle this complex dilemma, here, an HSE with rigid Li_1.5_Al_0.5_Ge_1.5_(PO_4_)_3_ (LAGP) core@ultrathin flexible poly (vinylidene fluoride-hexafluoropropylene) (PVDF-HFP) shell interface has been developed. The introduced large amount of nanometer-sized LAGP cores can not only act as structural enhancer to achieve high Young's modulus but can also construct Li^+^ diffusion network to homogenize Li^+^ distribution. The ultrathin flexible PVDF-HFP shell provides soft and stable contact between the rigid core and Li metal without affecting the Li^+^ distribution, meanwhile suppressing the reduction of LAGP induced by direct contact with Li metal. Thanks to these advantages, this ingenious HSE with ultra-high Young's modulus of 25 GPa endows dendrite-free Li deposition even at a deposition capacity of 23.6 mAh. Moreover, with the successful inhibition of Li dendrites, the HSE-based quasi-solid-state Li-O_2_ battery delivers a long cycling stability of 146 cycles, which is more than three times that of gel polymer electrolyte-based Li-O_2_ battery. This new insight may serve as a starting point for further designing of HSE in Li-O_2_ batteries, and can also be extended to various battery systems such as sodium-oxygen batteries.

## Teaser

Hybrid solid electrolyte with rigid core@ultrathin flexible shell interface suppresses Li dendrite formation effectively, enabling high-performance quasi-solid-state lithium-oxygen batteries.

## INTRODUCTION

Recently, lithium-oxygen (Li-O_2_) batteries have attracted wide attention because they have 10 times the theoretical energy densities of lithium-ion batteries. The Li-O_2_ battery is composed of cathode, anode and electrolyte [1], among which, the widely used organic electrolyte (OE) is the most urgently in need of improvement owing to its security risk caused by Li dendrite and poor thermal safety [2–4]. Considering the high thermal stability and Li dendrite suppression ability of solid-state electrolyte, it has been regarded as the best alternative to OE in figuring out the issues of OE encountered [5].

The commonly used solid-state electrolyte can be broadly classified into two types: ceramic electrolyte (CE) and polymer electrolyte (PE). Although CE can offer good thermal stability, high Young's modulus and single ion conduction, the uneven contact between rigid CE and electrode hinders its successful use to produce high-performance batteries [6–9]. However, this is not a problem for PE because PE possesses outstanding flexibility and good wetting ability towards electrodes. What is more, the gel polymer electrolyte (GPE) obtained by adding plasticizer to PE holds good ionic conductivity and stability with Li anode [10,11]. As a result, poly (vinylidene fluoride-hexafluoropropylene) (PVDF-HFP)-based GPE, a representative GPE, has been widely used in Li-O_2_ batteries due to its good stability and processability compared to other GPEs [12,13]. Unluckily, the disordered ion distribution and the intrinsic softness of the GPE make it useless in stopping Li dendrite formation during long-term cycling [14,15]. Since both CE and GPE alone cannot meet all the requirements of a perfect solid-state electrolyte, naturally, hybrid solid electrolyte (HSE), which can combine the advantages of CE and GPE, is promising to further promote the performance of the electrolyte, and has received extensive interest.

Although there are some HSEs that have been used in Li-O_2_ batteries, the ability of these HSEs for inhibiting dendritic Li growth is still poor due to their negligence of modulus and nonuniform Li^+^ distribution [16–20]. It is widely accepted that high Young's modulus and homogeneous Li^+^ distribution are the key points in realizing dendrite-free Li deposition [21,22]. Therefore, to achieve dendrite-free Li deposition in the semi-open system of Li-O_2_ batteries, HSE must meet the following conditions simultaneously: (i) the introduced rigid CE with three-dimensional ion transport channels must cover the whole surface of Li metal to ensure uniform Li^+^ distribution and high modulus [23,24], (ii) the HSE should be soft and smooth enough to ensure good contact with Li metal, (iii) the distribution of GPE and CE in the HSE should be homogeneous to avoid local disorder of Li^+^ [25–28], and (iv) the HSE should be O_2_ or air stable [29]. Among all the available CEs, Na^+^ superionic conductor (NASICON) type CE, represented by Li_1.5_Al_0.5_Ge_1.5_(PO_4_)_3_ (LAGP), is the best candidate for constructing HSE because of its high stability in air. However, the LAGP cannot contact Li metal directly due to the reduction of Ge^4+^ by Li metal. Hence, a stable interface layer should be built between the Li metal and LAGP in HSE [30–33]. It is no doubt that there are contradictions among these requirements. Therefore, how to rationally design HSE to tackle the complex dilemma is difficult but necessary.

**Figure 1.**
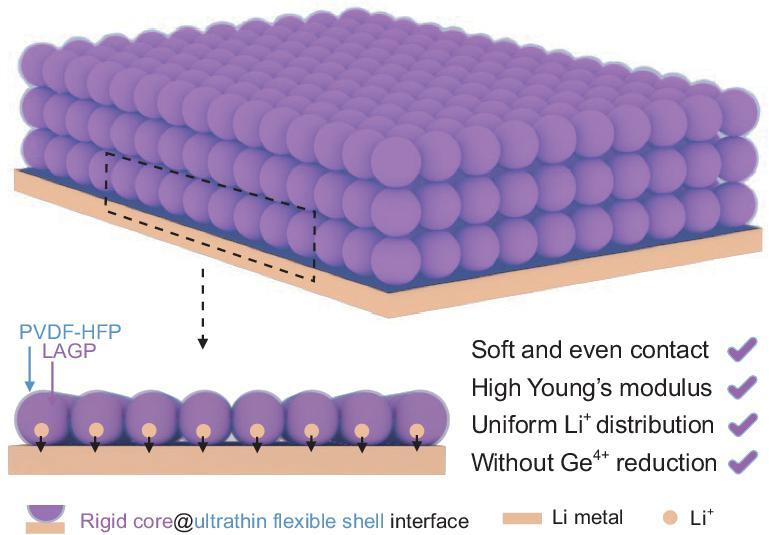
Schematic representation of dendrite-free lithium deposition enabled by HSE.

To meet the above requirements concurrently, herein an HSE with rigid core@ultrathin flexible shell interface has been designed by simply mixing nanometer-sized LAGP and a small amount of PVDF-HFP (Fig. S1a). As illustrated in Fig. 1, the low content of PVDF-HFP makes it form an ultrathin flexible shell on the surface of the rigid LAGP core. This ultrathin flexible shell can provide soft contact between the LAGP core and Li metal without affecting the Li^+^ distribution near the Li metal, and suppress the Ge^4+^ reduction induced by direct contact with Li metal. The large number of nanometer-sized LAGP cores ensures that the entire Li metal surface is in close contact with the CE, providing uniform distribution of Li^+^ and high Young's modulus. Based on this unique core@shell interface of HSE, dendrite-free Li deposition has been achieved both in symmetric batteries and quasi-solid-state Li-O_2_ batteries, as a result, prolonging the cycling life of the quasi-solid-state Li-O_2_ battery.

## RESULTS AND DISCUSSION

The ratio of PVDF-HFP/LAGP and the size of LAGP used in preparation of HSE have a strong impact on the core@shell interface. Therefore, we have set up three comparison groups (HSE-I with high-content, micro-sized LAGP; HSE-II with low-content, nanometer-sized LAGP; GPE without LAGP) to study how the core@shell interface works.

**Figure 2.**
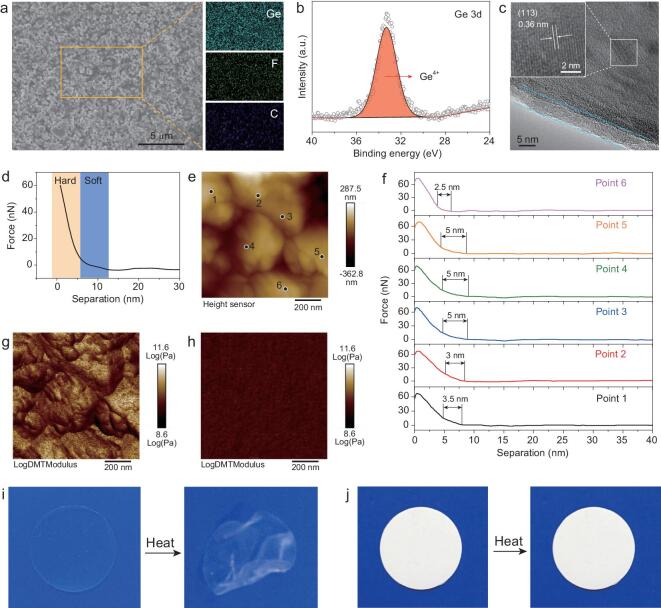
(a) Scanning electron microscope (SEM) image of HSE and the corresponding energy dispersive spectroscopy (EDS) mapping images of Ge, F and C elements. (b) Ge 3d XPS spectrum of HSE. (c) HRTEM image of HSE. (d) The force-separation curve of HSE. (e) AFM topography of HSE and (f) the corresponding force-separation curves of six points. Young's modulus mapping of (g) HSE and (h) GPE. Digital pictures of (i) GPE and (j) HSE before and after heating at 150°C for 5 min.

As shown in Fig. 2a, the surface of HSE is relatively flat and smooth. This advantage allows HSE to maintain a close and even contact with the electrode. The homogeneous distribution of the Ge element of LAGP, and the C and F elements of PVDF-HFP confirms that the LAGP and PVDF-HFP are mixed uniformly. Besides, the HSE has excellent flexibility (Fig. S1b), making it adapt well to the volume change of the electrode and be a promising candidate for constructing flexible quasi-solid-state batteries [34,35]. As indicated in Fig. S1c, the characteristic X-ray diffraction (XRD) peaks of the HSE are almost identical to those of LAGP powder, indicating that no undesirable reactions occurred during the solution-casting preparation process of the HSE. This can be further confirmed by the X-ray photoelectron spectroscopy (XPS) measurement (Fig. 2b), which shows that there is no Ge state change in the preparation process of the HSE, confirming the feasibility of the solution-casting process (Fig. S2) [36]. For identification of the detailed structure of HSE, high resolution transmission electron microscopy (HRTEM) was performed. Figure 2c reveals that the LAGP core is wrapped by an ultrathin PVDF-HFP shell with a thickness of ∼5 nm. It is clear that the lattice fringes of LAGP display an interplanar spacing of 0.36 nm, matching well with the (113) plane of LAGP.

The thickness of the PVDF-HFP shell was further detected by atomic force microscopy (AFM). The force-separation curve consists of two parts with different slopes, indicating that the probe touches two different substances (Fig. 2d). The arc curve at the beginning of deformation corresponds to the typical deformation character of a soft sample, such as polymer. Then the curve changes from an arc to a straight line, suggesting that the probe touches hard LAGP. Therefore, the force-separation curve demonstrates that the surface of the LAGP is covered by an ultrathin layer of soft polymer. To prove that the LAGP core is completely wrapped by the PVDF-HFP shell, six more points were chosen on HSE to test their force-separation curves (Fig. 2e). As displayed in Fig. 2f, all these six points show similar curves with an arc and a straight line, which confirms the uniformity and integrity of the core@shell interface of the HSE. Moreover, the force-separation curves also show that the thickness of the PVDF-HFP shell is around 5 nm, consistent with the HRTEM results. Electrochemical impedance spectra (EIS) measurement was then performed on HSE and LAGP pellet-based symmetric Li/Li batteries to check whether this ultrathin PVDF-HFP shell could improve the contact between the LAGP and Li metal electrodes (Fig. S3). The ionic conductivity of the LAGP pellet is 5.45 × 10^−4^ S cm^−1^ (Fig. S4), which is comparable to the reported result [33]. As shown in Fig. S5 and Table S1, with the introduction of the ultrathin shell, the interface resistance decreases by 185 times, indicating that the designed HSE could effectively improve the interfacial contact with the Li metal electrode, which is indispensable for guaranteeing the high performance of batteries.

By modulating the content and size of LAGP, we found that the morphology of the electrolyte changed significantly. Different to HSE, the surface of HSE-I is rough and rugged (Fig. S6), which would cause uneven contact between HSE-I and electrodes. AFM topography also confirms the more undulating surface of HSE-I than that of HSE (Figs 2e and S7), indicating that nanometer-sized LAGP can enable HSE with smooth surface and thus a good contact with electrodes. For HSE-II and GPE, smooth surfaces like HSE are observed (Fig. S8a and c). However, the main part of the HSE-II is PVDF-HFP, making the small portion of the LAGP form a discontinuous ion diffusion network, resulting in local disorder of Li^+^ and poor modulus. In view of the mechanical strength of electrolyte being critical to suppress Li dendrite growth, the Young's modulus property of the HSE has been tested by AFM [37]. The mean Young's modulus value of the HSE is 25 GPa (Fig. 2g), which is the highest value in the reported hybrid solid electrolyte and even exceeds the values of those artificial inorganic interface layers (Table S2). In contrast, the GPE exhibits a mean Young's modulus value as low as 4.2 GPa, which is too soft to suppress Li dendrite growth (Fig. 2h) [38]. Figure 2i and j shows the digital pictures of the GPE and HSE, respectively. The GPE is transparent and colorless, while the HSE displays uniform white color due to the existence of numerous white nanometer-sized LAGP. Since the thermal safety is critical for Li-O_2_ batteries, heat resistance tests for GPE, HSE, HSE-I and HSE-II have been performed. After being heated at 150°C for 5 minutes, the GPE is not flat anymore and exhibits a serious distortion (Fig. 2i). As indicated in Fig. S9, the HSE-I and HSE-II have obviously better thermal stabilities than that of GPE, but they still show some morphology change, indicating that the introduction of LAGP can to some extent improve the thermal stability of hybrid solid electrolyte. It is encouraging to note that the HSE experiences no shrinkage or decomposition after heat treatment (Fig. 2j), demonstrating its outstanding thermal stability. The higher thermal stability of  HSE compared to that of HSE-I and HSE-II reveals that the thermal stability of hybrid solid electrolytes is closely related to the content and size of LAGP [39].

Considering that the ionic conductivity of electrolyte has a significant effect on the polarization of batteries, EIS of the synthesized electrolytes have been tested (Fig. S10). It is well known that the ionic conductivity is a product of Li^+^ concentration and mobility, therefore the larger the content and the smaller the size of LAGP in the hybrid solid electrolyte, the higher the ion conductivity which can be obtained [40,41]. As a result, the HSE shows the highest ion conductivity, followed by HSE-I and HSE-II. The slightly lower ion conductivity of HSE-II compared to that of GPE is probably due to the relative rougher surface of HSE-II compared with GPE, making the transfer of Li^+^ encounter more resistant. In addition to ionic conductivity, the electrochemical stability of electrolyte is also an important character for batteries to achieve good performance. As shown in Fig. S11, the HSE manifests a remarkably enhanced electrochemical oxidation window up to 5.2 V, which meets the requirement of Li-O_2_ batteries. The worst electrochemical stability of HSE-I can be ascribed to its uneven and rugged surface, decreasing the effective contact area with Li metal. The large amount of PVDF-HFP in HSE-II makes it exhibit less stability than HSE, but HSE-II is still more stable than GPE due to the introduction of rigid LAGP.

In order to directly observe the effect of synthesized electrolytes on the morphology of lithium deposition, the Li-plating process was visualized by *insitu* optical microscopy. As shown in Fig. S12, Li dendrites rapidly nucleate and grow on the bare Li surface with plating time increasing. Although no dendrites can be detected on the GPE protected Li sheet at the beginning of deposition, Li dendrites emerge just after prolonging the plating time to one hour, and ultimately pierce the GPE as the deposition time increases. Dendrites are also formed on HSE-I and HSE-II protected Li sheets, but the morphology of the dendrites is different. Only a few dendrites appear on the HSE-I protected Li sheet, while the surface of the HSE-II protected Li sheet is completely covered by dendrites, probably due to the poor modulus induced by the insufficient amount of LAGP. In contrast, there is no dendrite forming on the HSE protected Li sheet during the whole plating process, indicating the effectiveness of the designed HSE for realizing dendrite-free Li deposition.

**Figure 3.**
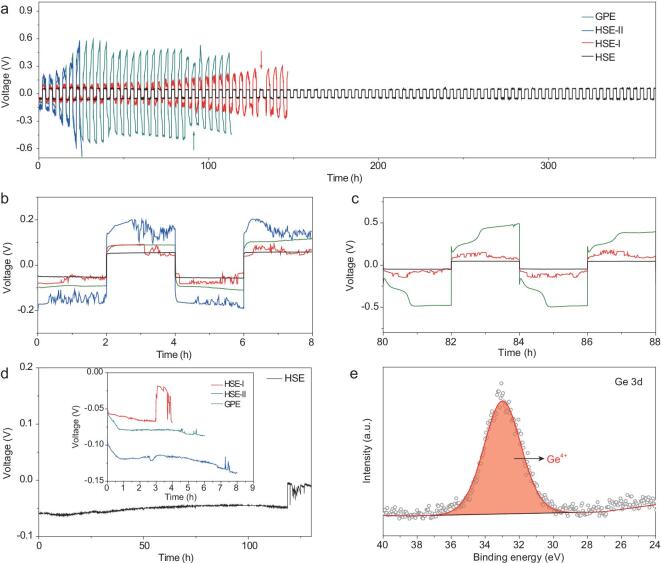
(a) Cycle life for symmetric Li/Li batteries with different electrolytes (current: 200 μA; each charge-discharge cycle length: 4 h). The arrows indicate short circuit. The magnified voltage profiles of (b) 0–8 h and (c) 80–88 h. (d) Li^+^ depletion time tests in symmetric Li/Li batteries at 200 μA. (e) Ge 3d XPS spectrum of HSE disassembled from symmetric Li/Li battery after 30 cycles.

To further confirm the validity of the HSE in suppressing Li dendrite growth, symmetric Li/Li batteries with the synthesized electrolytes were built. It can be seen from Fig. 3a that the Li/Li battery with HSE shows the longest cycle life without obvious increase in voltage hysteresis. This excellent performance can be attributed to the high ion conductivity and dendrite-free Li deposition behavior endowed by the HSE. For HSE-I, the cycle life is much less than that of HSE, revealing that even and intimate contact between core@shell interface and Li metal is important for enabling long-term cycling stability. More interestingly, although HSE-II contains LAGP, its cycle life is even shorter than that of GPE. Therefore, the distribution of core@shell interface near the Li surface has a big effect on suppressing dendrite growth. As shown in the partially magnified voltage curves (Fig. 3b and c), the HSE with a relatively flat voltage plateau always exhibits the smallest polarization. Although the polarizations of the GPE and HSE-I are almost same at the beginning of cycling, the polarization of the GPE increases rapidly as the cycle goes on, which means the fast growth of Li dendrites. In contrast, the polarization of  HSE-I manifests a much slower increase behavior, indicating that the growth of Li dendrites can be inhibited to some extent by its relatively high Young's modulus. For HSE-II, its poorest ion conductivity and low Young's modulus make it exhibit the largest polarization and shortest lifetime induced by the fast dendrite growth. Notably, except for HSE, the voltage curves of all the other electrolytes display obvious voltage fluctuation during cycling, implying unstable and non-uniform ion transfer. There is a slight difference in the voltage curves of the HSE-I and HSE-II. During each two hours of deposition, the voltage curve of the HSE-I begins to fluctuate at the later stage of deposition, while the voltage curve of the HSE-II fluctuates from the beginning of deposition. This phenomenon indicates that the ion transfer of HSE-I is uniform and stable at the beginning, but turns to non-uniform and unstable as time increases, while the ion transfer of HSE-II is non-uniform and unstable all the time.

The causes of the long stability of the battery with HSE can be further identified by testing the Li^+^ depletion time of the synthesized electrolytes-based symmetric Li/Li batteries. As shown in Fig. 3d, the Li^+^ depletion time is determined by where the voltage curve apparently diverges due to the fast Li dendrite growth induced by complete Li^+^ depletion [42]. The Li^+^ consumption time of the synthesized electrolytes follows the sequence of HSE-I<GPE<HSE-II<HSE. The shortest Li^+^ depletion time of HSE-I can be ascribed to its uneven contact with Li sheet, which would cause local Li^+^ to be quickly depleted. Although the Li^+^ depletion time decreases with the increase of the deposition current, the HSE always shows the longest depletion time and can still maintain 6 hours of stable Li plating at a high current of 500 μA (Figs S13 and S14), confirming that the designed HSE can effectively relieve Li^+^ depletion and inhibit Li dendrite growth. The strong dendrite inhibition ability of HSE can be further confirmed by the smooth and dendrite-free surface of Li sheet disassembled from symmetric HSE-based cells even at an ultra-high deposition capacity of 23.6 mAh. In contrast, Li sheets dissembled from symmetric Li/Li batteries with HSE-I/HSE-II/GPE are completely covered by Li dendrite even at lower deposition capacities (Figs S15–S17). In addition to Li^+^ depletion, the chemical stability of  LAGP is also worthy of attention. As shown in Fig. S18, when directly contacting the LAGP pellet with Li metal, two new peaks appear at 30 eV and 31.9 eV in the Ge 3d XPS spectrum, which can be attributed to Ge^0^ and Ge^2+^, respectively. In contrast, although the HSE endures 120 hours of cycling in battery, still only the Ge^4+^ peak can be detected (Fig. 3e), demonstrating that the ultrathin PVDF-HFP shell can effectively separate the LAGP core from the Li anode and avoid the Li metal induced Ge^4+^ reduction.

**Figure 4.**
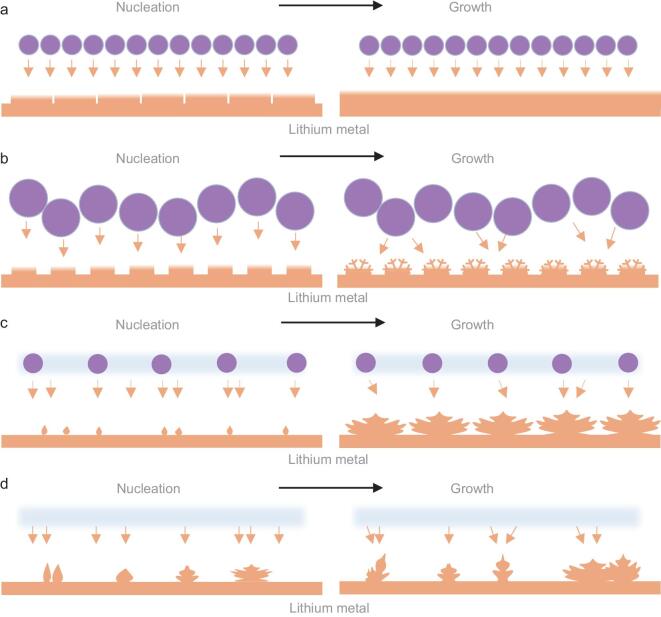
Schematic representation of Li deposition with (a) HSE, (b) HSE-I, (c) HSE-II and (d) GPE.

**Figure 5.**
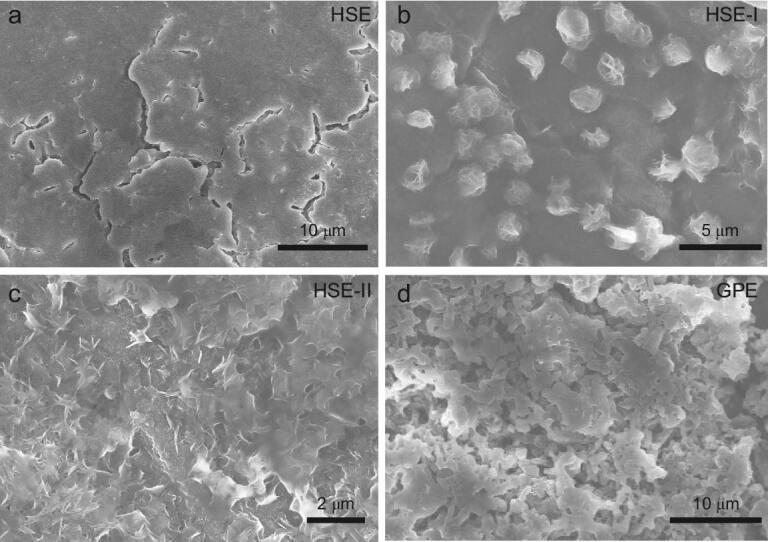
SEM images of Li sheets disassembled from symmetric batteries with (a) HSE, (b) HSE-I, (c) HSE-II and (d) GPE after cycling test.

The Li deposition mechanism with different electrolytes is illustrated in Fig. 4. The rigid core@ultrathin flexible shell interface provides HSE with uniform Li^+^ distribution, flexible and stable contact with Li sheet and high Young's modulus. Therefore, the Li^+^ deposits evenly at the nucleation and growth stages (Fig. 4a). For HSE-I, the distribution of Li^+^ is homogeneous initially, but the rough and rugged surface of HSE-I makes the ions in the locally uncontacted area with Li sheet quickly deplete, and as a result, the Li^+^ deposits uniformly at the nucleation stage and dendrites appear in the later growth stage (Fig. 4b). The local disorder of Li^+^ induced by the insufficient amount of LAGP in the HSE-II causes dendrite formation at the nucleation stage (Fig. 4c). As the deposition progresses, the local uneven distribution of Li^+^ would be deepened and Li^+^ would become less or be completely depleted, resulting in the rapid growth of a large number of dendrites. Although there is a good contact interface formed between the GPE and Li sheet, the uneven distribution of Li^+^ results in the generation of dendrites, and the continuously formed dendrites will finally pierce into the soft GPE (Fig. 4d). In order to further verify our view, the morphology evolution of the different electrolytes protected lithium sheets after the cycling test was investigated by SEM (Fig. 5). Except for some cracks, no lithium dendrite can be discerned on the surface of the Li sheet taken from failed HSE-based battery (Fig. 5a). The cracks induced by the infinite volume change of Li during cycling may be the root of battery performance deterioration [43]. Although the HSE-I cannot completely rule out dendrite growth, only a small amount of sphere dendrites (later deposition) are produced on the initially formed dense Li layer (early deposition). Therefore, the cycle life of symmetric HSE-I-based battery is second to that of HSE-based battery (Fig. 5b). The ultrathin thickness of the dendrites on the HSE-II protected Li sheet surface leads to a strong tip electrical field, which would aggravate the dendrite growth. This is also an important reason for the worst performance of the HSE-II-based symmetic cell (Fig. 5c) [44]. For GPE-based battery, large and loose dendrites cover the Li sheet surface (Fig. 5d). These results demonstrate that the Li deposition behavior and the final morphology of lithium dendrites for the synthesized electrolytes are consistent with our expectations.

**Figure 6.**
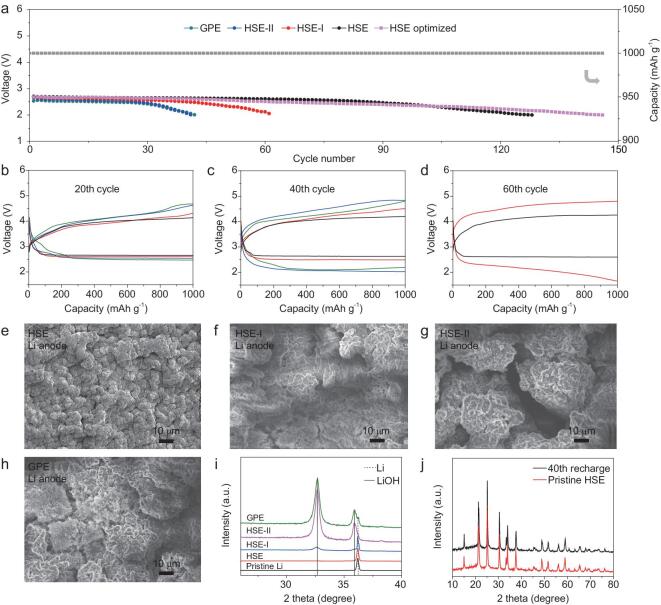
(a) The cycle life of quasi-solid-state Li-O_2_ batteries with different electrolytes. The discharge-charge curves of quasi-solid-state Li-O_2_ batteries at (b) 20th, (c) 40th and (d) 60th cycle. (e–h) SEM images and (i) XRD patterns of the Li anodes disassembled from quasi-solid-state Li-O_2_ batteries after 40 cycles. (j) XRD patterns of the HSE before and after 40 cycles.

Inspired by the successful dendrite control through HSE with rigid core@flexible shell interface, HSE-based quasi-solid-state Li-O_2_ batteries with Ru decorated-carbon nanotube (Ru-CNT) cathodes were assembled (Fig. S19). Figure 6a shows the discharge terminal voltage vs. cycle number profiles of the quasi-solid-state Li-O_2_ batteries with different electrolytes cycled at a cutoff capacity of 1 000 mAh g^−1^ and a current density of 300 mA g^−1^. Considering that the discharge capacity of carbon paper is quite limited [45], here, the capacity and current density are all based on the loading amount of active material: painted Ru-CNTs. It is clear that the HSE-based battery can realize 128 times stable cycling before the discharge terminal voltage reaching 2 V. In contrast, the discharge terminal voltages of the HSE-I, HSE-II and GPE-based batteries degrade to 2 V after only 60, 41 and 42 cycles, respectively. Since the loading amount of active materials has a significant effect on the battery life, the life of the HSE-based battery can be extended from 128 to 146 cycles by optimizing the loading amount of Ru-CNTs from 0.35 to 0.2 mg cm^−2^. This improved life is comparable or superior to the reported works (Table S3). In order to figure out the impact of Li anodes on the quasi-solid-state Li-O_2_ batteries during cycling, the discharge-charge curves at different cycles are shown in Fig. 6b–d. Regardless of the number of cycles, the HSE-based quasi-solid-state Li-O_2_ batteries always exhibit the smallest charging (Li deposition process) over-potential and show no significant increase in over-potential as the cycle goes on, which is in sharp contrast to the other three electrolytes with rapid increase in charging over-potentials during cycling. The over-potential increase speed follows the sequence of HSE-II>GPE>HSE-I>HSE, which is consistent with the over-potential growth trend in symmetric Li/Li batteries. Suppressing dendrite growth can reduce the exposure of fresh lithium and then improve the anti-corrosion ability of Li sheets [46,47]. Therefore, the strong dendrite suppression ability of the HSE prolongs the lifetime of the quasi-solid-state Li-O_2_ batteries. To visually observe the evolution of Li anodes in the quasi-solid-state Li-O_2_ batteries, the morphology of Li anodes after 40 cycles was examined [48,49]. Unlike the initial smooth surface (Fig. S20), the morphology of the Li anodes changes after cycling. It can be seen from Fig. 6e that the Li anode in the HSE-based battery is still relatively dense and flat. On the contrary, the Li anodes in HSE-I, HSE-II and GPE-based batteries exhibit loose and porous structures and the morphology of the Li surface gets worse in the order of HSE-I, GPE and HSE-II (Fig. 6f–h). These loose and porous Li anodes hinder the transport of Li^+^ and reduce the effective contact area with the electrolytes, thus increasing the impedance of the batteries and finally leading to the early death of the batteries. To further understand the underlying reasons for the above changes for Li anodes, the composition evolution of the cycled Li metals were examined by XRD. As shown in Fig. 6i, the Li anode in the HSE-based battery exhibits much lower LiOH peak intensity than that in other electrolytes-based batteries, revealing the least exposure surface of Li endowed by HSE. It is clear that the intensity of the LiOH peak is inversely proportional to the ability of the electrolyte to suppress dendrites. Considering that the amount of LiOH on the Li metal surface can behave as an index to identify the corrosive degree of Li metal, these above findings substantially confirm that the suppression of Li dendrites can effectively alleviate Li corrosion, thereby improving battery performance. To explore the formation-decomposition processes of discharge products in quasi-solid-state Li-O_2_ batteries with HSE, XRD and SEM were performed on the cathodes at different states. As shown in Fig. S21, only the peaks associated with Li_2_O_2_ are observed after discharging and these peaks disappear after subsequent recharging. Besides, it can be clearly seen that toroidal-like discharge products are well distributed along the Ru-CNTs (Fig. S22). After charging, these discharge products decompose completely. Therefore, HSE does not affect the reaction mechanism of Li-O_2_ batteries which follows a typical Li_2_O_2_ formation-decomposition pathway. What is more, HSE shows robust electrochemical stability which can be proved by the following SEM and XRD tests. As shown in Fig. S23, the HSE well maintains the compact morphology of the pristine HSE without any physical deterioration after 40 cycles, revealing the robustness of the HSE to withstand the rigorous cycling environment of a Li-O_2_ battery. Besides, the corresponding XRD pattern of the cycled HSE is accordant with the pristine state, further testifying the excellent stability of HSE in the harsh oxidation environment during the cycling of a Li-O_2_ battery (Fig. 6j).

Nowadays, electronics play an important role in our daily life. As the energy supply of those electronics, the safety of batteries should not be underestimated. Here, a cable-type HSE-based quasi-solid-state Li-O_2_ battery has been assembled to check its heat resistance ability. As displayed in Fig. S24, even after heating and bending, this cable-type battery can still work normally on powering the red light-emitting diode display screen, indicating that the HSE can successfully deal with the safety problems caused by thermal runaway. In contrast, the battery with GPE is short-circuited after heating due to the unsatisfactory thermal stability of GPE, highlighting the importance of a heat resistant electrolyte.

## CONCLUSION

In summary, an ingenious HSE with rigid core@ultrathin flexible shell interface has been designed to realize dendrite-free Li deposition. In the HSE, each of the nanometer-sized LAGP core is wrapped by a soft PVDF-HFP shell with a thickness of ∼5 nm. The ultrathin PVDF-HFP flexible shell provides soft and stable contact between the LAGP core and Li metal without affecting the Li^+^ distribution near the Li sheet, and suppresses the reduction of LAGP core by Li metal. The large number of nanometer-sized LAGP cores afford uniform distribution of Li^+^ and high Young's modulus. The synergistic interaction of the rigid LAGP core and the soft PVDF-HFP shell achieves excellent physical and chemical stability, uniform Li^+^ distribution near Li metal, ultra-high Young's modulus of 25 GPa and good contact with electrodes, leading to dendrite-free Li deposition with a large deposition capacity up to 23.6 mAh. As a proof of concept application, the quasi-solid-state Li-O_2_ battery with HSE shows a long cycle life of 146 cycles, and works normally even after heating treatment. This new insight may serve as a starting point for further design of hybrid solid electrolytes for Li-O_2_ batteries, and can also be extended to various battery systems such as sodium metal batteries by substituting ion conductors.

## METHODS

### Preparation of HSE, HSE-I, HSE-II and GPE

Preparation of HSE was as follows: 0.7 g nanometer-sized LAGP powder was first well distributed in N-methyl-2-pyrrolidinone (NMP) under ultrasonic, then 0.3 g PVDF-HFP was added under stirring to obtain a stable sticky mixture. After this, the obtained mixture was casted on a glass plate by a doctor blade, and finally dried in a vacuum oven at 70°C for 24 h to obtain HSE membrane. The preparation process of HSE-I was similar to that of HSE, except nanometer-sized LAGP were changed to micro-sized LAGP. The same process as that of HSE was used to prepare HSE-II, except the ratio of nanometer-sized LAGP to PVDF-HFP was changed from 7 : 3 to 3 : 7. The preparation process of GPE was similar to that of HSE, except without the addition of LAGP.

### Characterizations

The material morphology was examined using a Hitachi S-4800 FE-SEM operating at 10 kV. Powder XRD tests were performed on a Bruker D8 Focus powder X-ray diffractometer using Cu Kα radiation. The tapping mode of AFM (Bruker Dimension Icon) was used to measure the morphology of samples. The mechanical properties mode of AFM was employed to test the force-separation curve and Young's modulus of samples. The probe model was RTESP-MPP-11100-10. Deflection sensitivity was 40.476 nm V^−1^. Spring constant (k) was 50.4 N m^−1^. Amplitude was 50 nm. f_0_ was 327 kHz. Tip radius was 15.3 nm. EIS was tested with frequency ranging from 10^6^ to 10^−1 ^Hz at AC amplitude of 10 mV. The results were analyzed by Zview software. Linear sweep voltammetry (LSV) tests were measured on a BioLogic VMP3 electrochemical workstation with a scan rate of 1 mV s^−1^. Transmission electron microscopy (TEM) was performed using an FEI Tecnai G2 S-Twin transmission electron microscope with a field emission gun operating at 200 kV. The stability of nanometer-sized LAGP against Li metal was checked by pressing a Li sheet on the surface of a LAGP pellet for 5 days. Then XPS analyses were carried out to check the surface of the LAGP pellet on an ESCALAB MKII X-ray photoelectron spectrometer. Ionic conductivity σ was calculated as $\sigma = \frac{l}{{\,R\times\,S}}$, where *R* represents the resistance, *l* represents the thickness of the electrolyte and *S* is the area.

### Assembly of a quasi-solid-state Li-O_2_ battery and electrochemical measurements

The as-synthesized electrolytes were activated with OE (1 M lithium triflate in tretraethylene glycol dimethyl ether). Each of the 2025 type quasi-solid-state Li-O_2_ battery was assembled with Ru-CNTs cathode, activated quasi-solid-state electrolyte and Li anode (thickness: 0.4 mm; diameter: 14 mm) in an argon-filled glove box. All battery tests were carried out in the bottles full of pure O_2_ at normal atmospheric pressure. Electrochemical performance measurements of the quasi-solid-state Li-O_2_ cells were conducted on a LAND CT 2001A multichannel battery testing system.

## Supplementary Material

nwaa150_Supplement_File
