# Supplementary material for: Hybrid solid electrolyte enabled dendrite-free Li anodes for high-performance quasi-solid-state lithium-oxygen batteries
Source: Natl Sci Rev. 2020 Jul 2;8(2):nwaa150. doi: 10.1093/nsr/nwaa150 (PMC8288355; doi:10.1093/nsr/nwaa150)
Supplement: nwaa150_Supplement_File [file nwaa150_supplement_file.docx]

Supporting Information

**Hybrid solid electrolyte enabled dendrite-free Li anodes for high-performance quasi-solid-state lithium-oxygen batteries**

**Preparation of** **micron sized LAGP (µm-****LAGP)**

Mixture of Li_2_CO_3_, Al_2_O_3_, NH_4_H_2_PO_4_ and GeO_2_ was first milled for 2 h at 400 rpm (Pulverisette 7, Fritsch), and then annealed at 900 ºC for 6 h.

**Preparation of LAGP pellet**

The nanometer sized LAGP powder was pressed into a pellet using a 12 mm diameter die and then sintered at 900 ºC for 8 h.

**Preparation of Ru-CNTs cathode**

0.6 g CNTs and 1 g Pluronic F127 were first dispersed in 300 mL distilled water under vigorous stirring for 24 h. Then, 600 mg RuCl_3_•xH_2_O was added into the above suspension. After vigorous stirring for another 24 h, the obtained mixture was dried in air at 100 ºC for 48 h and then heated at 300 ºC for 3 h in a tubular furnace under 5% H_2_/Ar atmosphere. Finally, the sample was washed with ethanol for several times and dried in air at 60 ºC. The as-obtained Ru-CNTs were mixed with poly(vinylidene fluoride) (PVDF) in a mass ratio of 6:1 and dispersed in NMP to form a slurry. The as-prepared slurry was painted onto a carbon paper and dried in a vacuum oven at 80 ºC to get the cathode. The mass loading of the cathode was about 0.35 mg cm^-2^.

**Assembly of cable-type flexible quasi-solid-state Li-O_2_ battery**

First, a copper wire was inserted into lithium rod (diameter: 6 mm. length: 5 cm) to serve as anodic current collector. Then, HSE mixture was covered on the lithium rod and dried at room temperature. After this, the HSE was activated by OE followed by winding Ru-CNTs cathode (width: 1 cm. length: 10 cm) on its surface. To ensure uniform diffusion of air in cathode, nickel foam was wound around the cathode to behave as air diffusion layer. This cable-type battery was finally packaged by heat shrinkable rubber cable with pores.


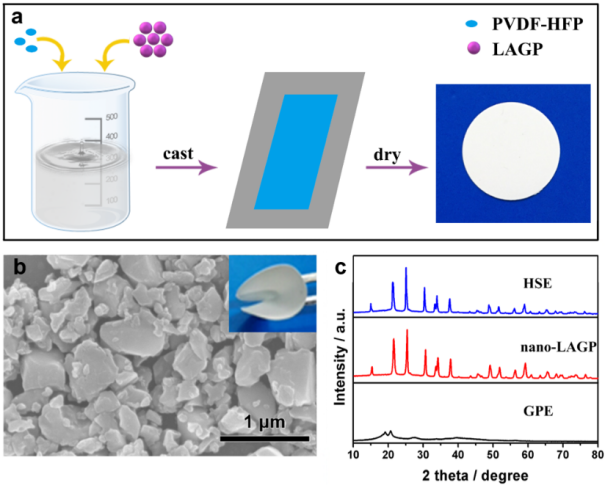


**Figure S1.** (a) Schematic representation for the preparation process of HSE. (b) SEM image of nanometer sized LAGP. Inset shows the flexibility of HSE. (c) XRD patterns of HSE, nanometer sized LAGP powder and GPE.


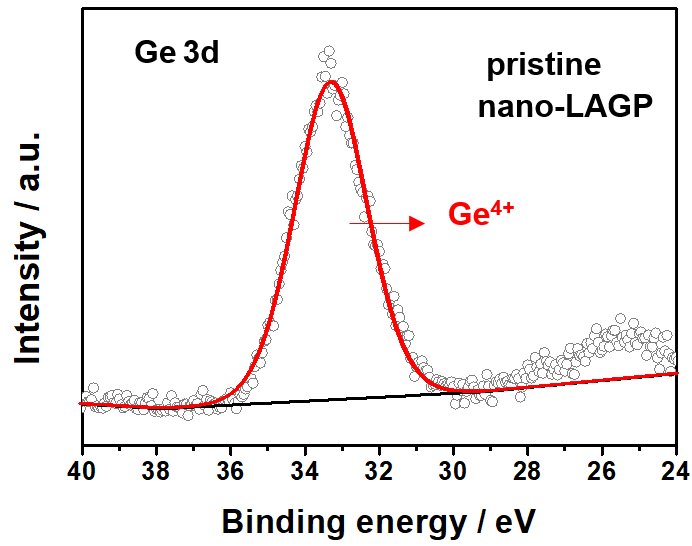


**Figure S2.** Ge 3d XPS spectra of pristine nanometer sized LAGP.

**
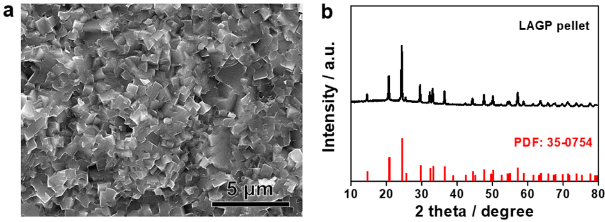
Figure S3.** (a) SEM image and (b) XRD pattern of the LAGP pellet.


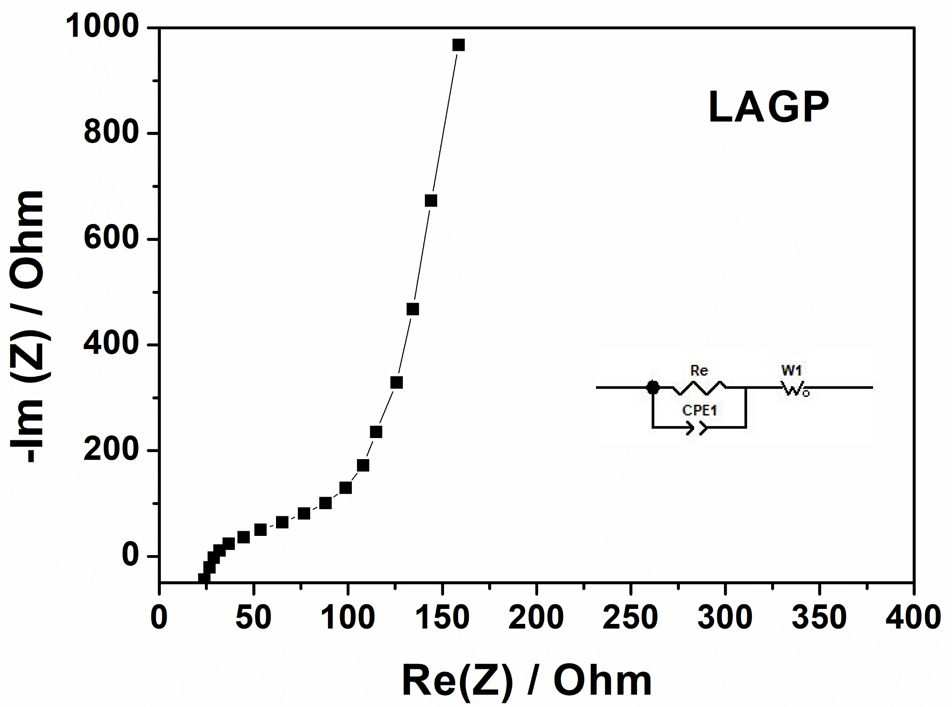


**Figure S4.** Electrochemical impedance spectrum (EIS) of the LAGP pellet. Inset is the equivalent circuit used to model the EIS response. The thickness and diameter of the LAGP pellet are 0.0984 cm and 1.1836 cm, respectively. The fitting value of Re is 164 Ω.


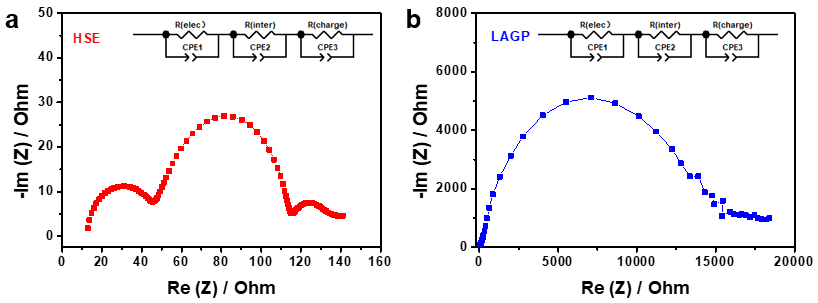


**Figure S5.** EIS of symmetric Li/Li batteries with (a) HSE and (b) LAGP pellet. Inset are the equivalent circuits. R (elec) is the electrolyte resistance. R (inter) is the electrolyte/Li interfacial resistance. R (charge) is the charge-transfer resistance.

**Table S1.** The fitting values of equivalent circuit elements in Figure S5.

| **Electrolyte** | **Electrolyte resistance (Ω)** | **Interfacial resistance (Ω)** | **Charge-transfer resistance (Ω)** |
| --- | --- | --- | --- |
| HSE | 67.36 | 63.87 | 29.2 |
| LAGP | 132 | 11844 | 7430 |


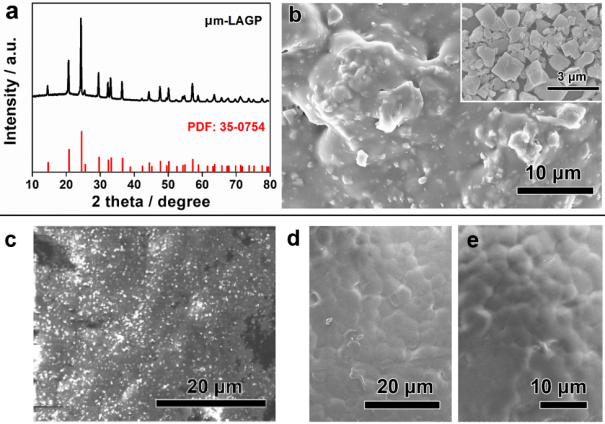


**Figure S6.** (a) XRD pattern of µm-LAGP. (b) SEM image of HSE-I. Inset is the SEM image of µm-LAGP.


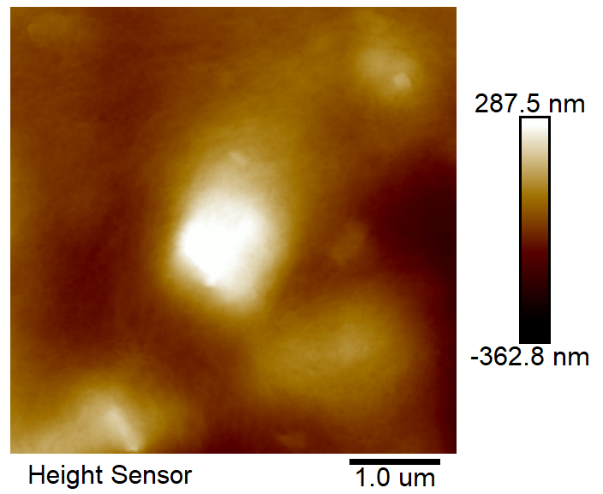


**Figure S7.** AFM topography of HSE-I.


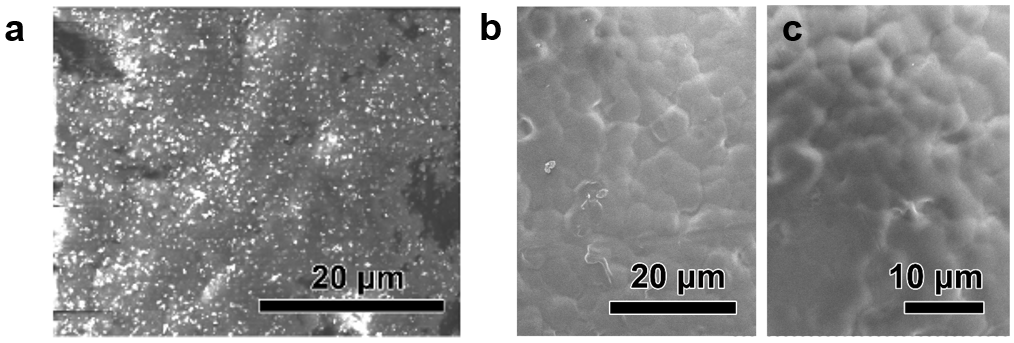


**Figure S8.** SEM images of (a) HSE-II and (b-c) GPE.

**Table S2.** Comparison of Young’s modulus values of hybrid solid electrolytes and solid electrolyte interphase layers.

| **Polymer** | **Inorganics** | **Young’s modulus** | **Ref.** |
| --- | --- | --- | --- |
| - | Li_3_PO_4_ | 10-11 GPa | 1 |
| PEO | LATP | 6.6 MPa | 2 |
| PVDF | LLZTO | 30.8 MPa | 3 |
| PVDF-HFP | LiF | 6.72 GPa | 4 |
| PVDF-HFP | LLZO | 12.5 MPa | 5 |
| PVDF-HFP | BN | 133 MPa | 6 |
| PEO | LLZO | 6.4 GPa | 7 |
| -(CO)_x_PO_x_- | Li_3_PO_4_ | 12 GPa | 8 |
| **PVDF-HFP** | **LAGP** | **25 GPa** | **This work** |


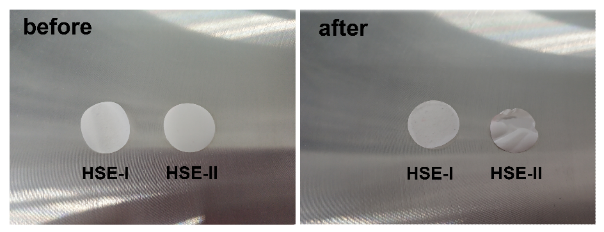


**Figure S9.** Digital pictures of HSE-I and HSE-II before and after heating at 150 ^o^C for 5 minutes.


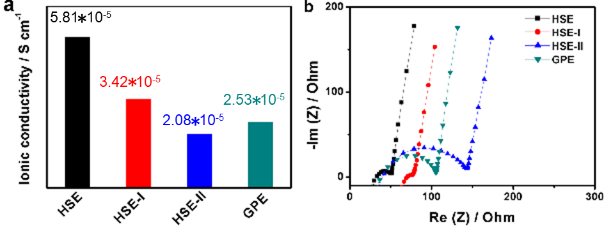


**Figure S10.** (a) Ion conductivity of different electrolytes. (b) Electrochemical impedance spectra of different electrolytes.


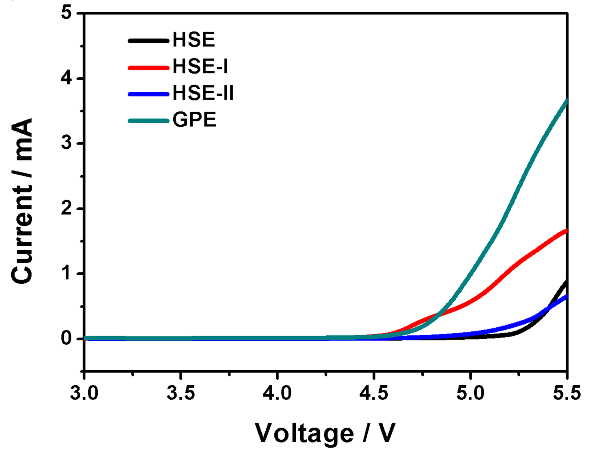


**Figure S11.** Linear sweep voltammetry curves of different electrolytes.


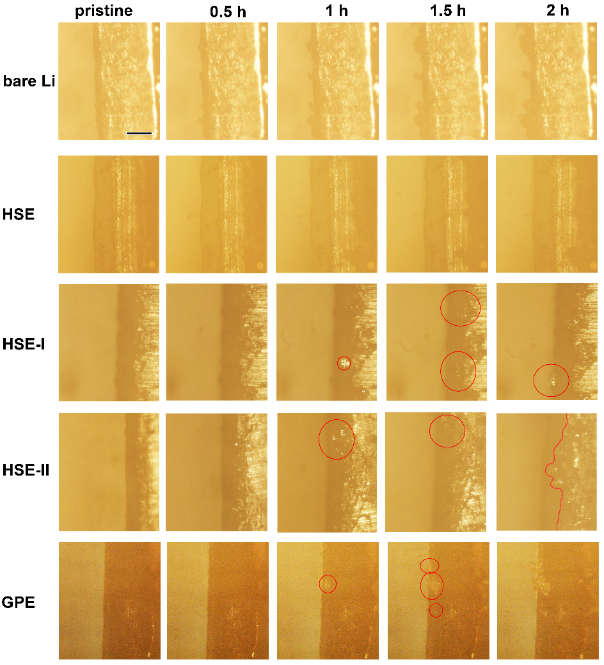


**Figure S12.** Snapshots of the Li deposition in a custom-made optical visualization battery that with bare Li, HSE protected Li, HSE-I protected Li, HSE-II protected Li and GPE protected Li electrodes (current: 100 µA cm^-2^). The red circle indicates Li dendrites. The scale bar is 100 µm and are same for all pictures.


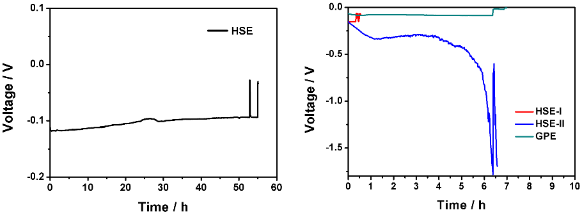


**Figure S13.** Li^+^ depletion time tests in symmetric Li/Li batteries with different electrolytes at a current of 300 µA.


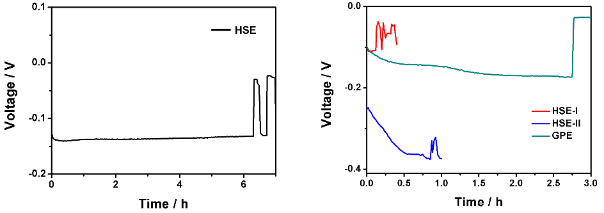


**Figure S14.** Li^+^ depletion time tests of symmetric Li/Li batteries with different electrolytes at a current of 500 µA.


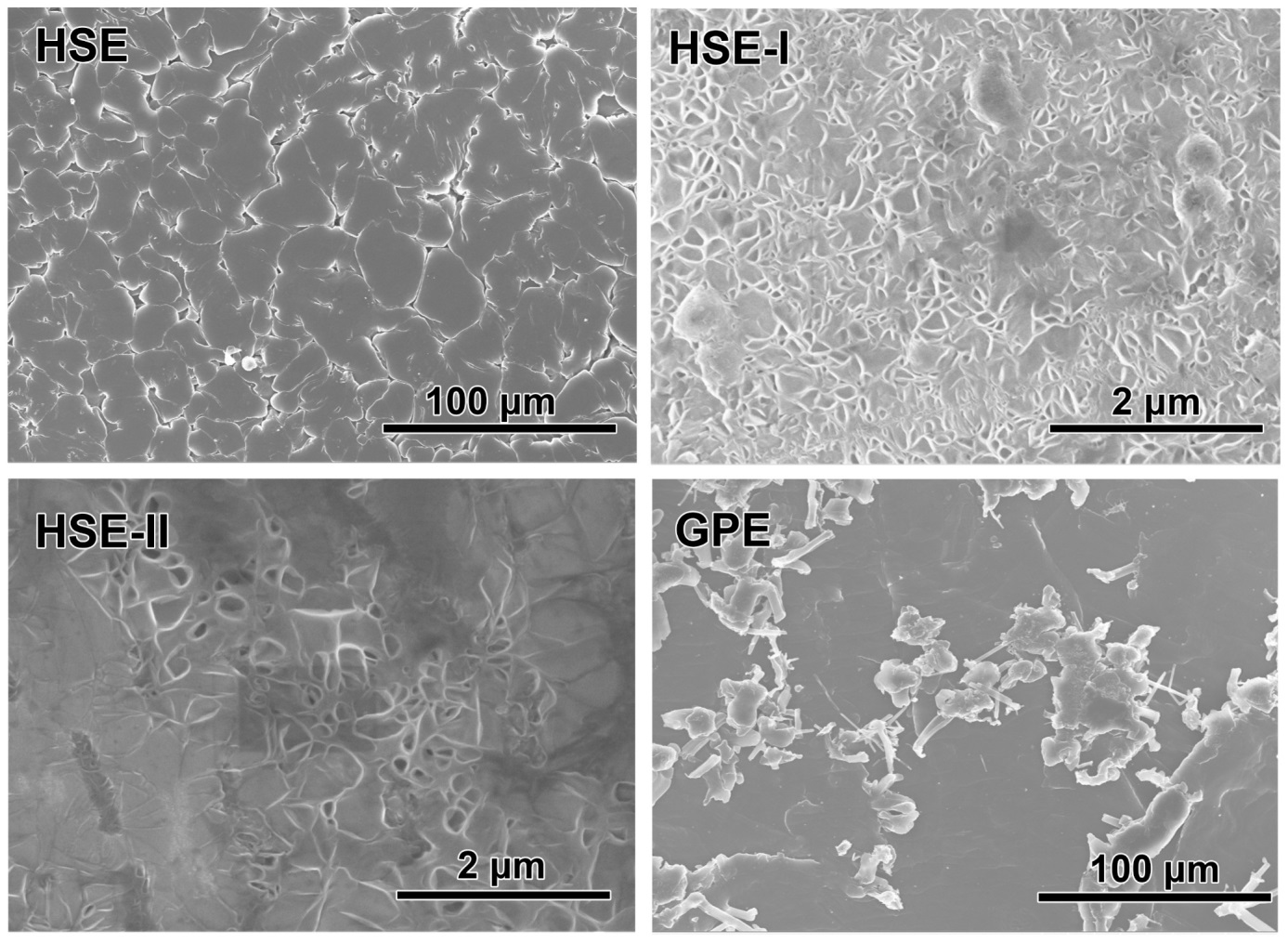


**Figure S15.** SEM images of Li sheets disassembled from symmetric Li/Li batteries after Li^+^ depletion time tests with different electrolytes at a current of 200 µA.


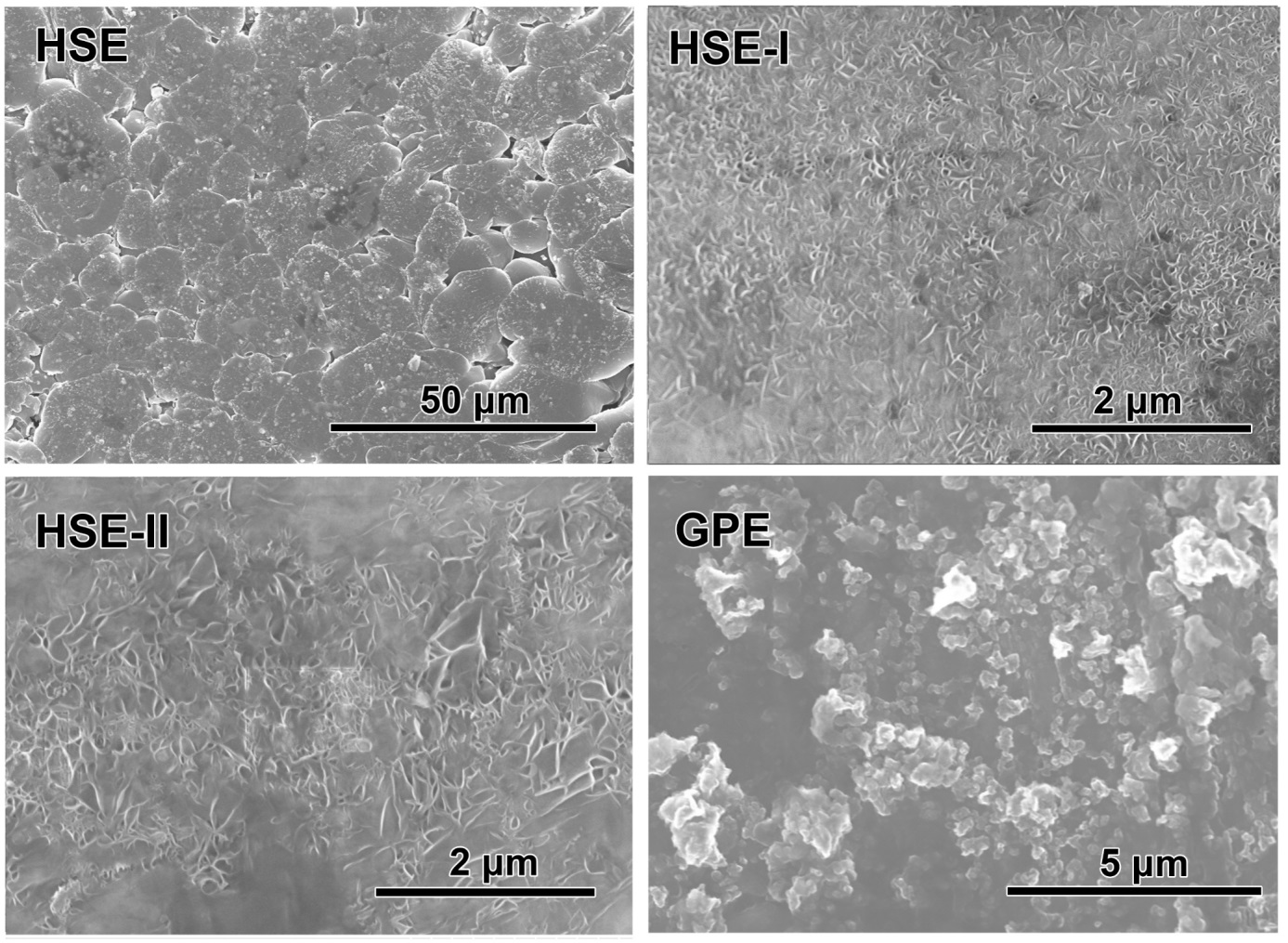


**Figure S16.** SEM images of Li sheets disassembled from symmetric Li/Li batteries after Li^+^ depletion time tests with different electrolytes at a current of 300 µA.


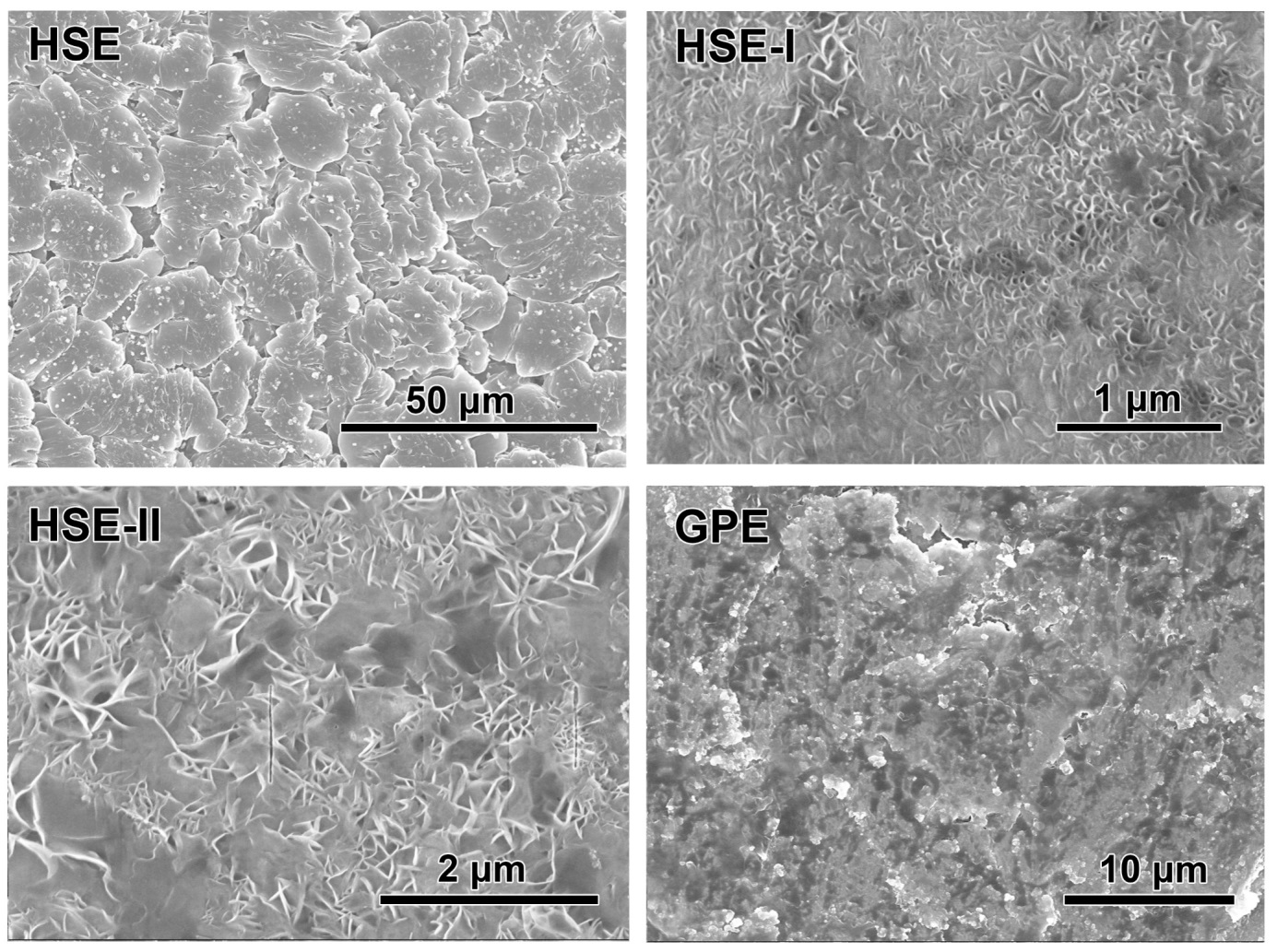


**Figure S17.** SEM images of Li sheets disassembled from symmetric Li/Li batteries after Li^+^ depletion time tests with different electrolytes at a current of 500 µA.


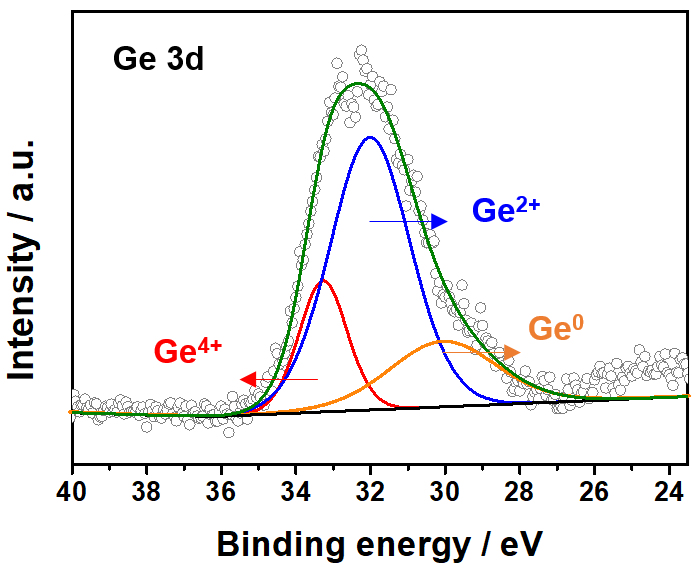


**Figure S18.** Ge 3d XPS spectra of LAGP pellet after contact with Li metal.

**
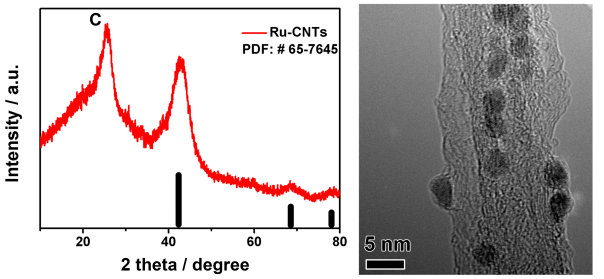
Figure S19.** XRD pattern and TEM image of Ru-CNTs.

**Table S3.** Comparison of quasi-solid-state Li-O_2_ batteries with different hybrid solid electrolytes.

| **Polymer** | **Inorganic filler** | **Plasticizer** | **Temperature/Atmosphere** | **Cathode** | **Performance** | | **Ref.** |
| --- | --- | --- | --- | --- | --- | --- | --- |
| PVDF-HFP | Al_2_O_3_ | TEGDME/LiClO_4_/TEMPO | Room temperature/O_2_ | Super-P | 156 mA g^-1^  600 mAh g^-1^  175 cycs | 9 | |
| P(MMA-st) | LiNbO_3_ | TEGDME/LiTFSI | 25 ºC/O_2_ | LSM@CNG | 250 mA g^-1^  1000 mAh g^-1^  100 cycs | 10 | |
| PEGMA | SiO_2_ | TEGDME/LiCF_3_SO_3_ | Room temperature/O_2_ | Pd_3_Co/CNT | 100 mA g^-1^  500 mAh g^-1^  125 cycs | 11 | |
| PVDF-HFP | Li_7_La_3_Zr_2_O_12_ | TEGDME/LiTFSI | Room temperature/O_2_ | Co_3_O_4_ | 400 mA g^-1^  500 mAh g^-1^  200 cycs | 12 | |
| Li-Nafion | Al_2_O_3_ | TEGDME/LiTFSI/TEMPO/DBBQ | Room temperature/O_2_ | CNT | 200 mA g^-1^  2000 mAh g^-1^  30 cycs | 13 | |
| TPU | Hydrophobic SiO_2_ | TEGDME/LiCF_3_SO_3_ | Room temperature/O_2_ | Super-P | 200 mA g^-1^  500 mAh g^-1^  114 cycs | 14 | |
| PVDF-HFP | Super-hydrophobic SiO_2_ | TEGDME/LiTFSI | Room temperature/O_2_ | Ru/ Super-P | 200 mA g^-1^  1000 mAh g^-1^  50 cycs | 15 | |
| **PVDF-HFP** | **LAGP** | **TEGDME/LiCF_3_SO_3_** | **32 ºC/O_2_** | **Ru-CNTs** | **300 mA g^-1^**  **1000 mAh g^-1^**  **146 cycs** | **This work** | |


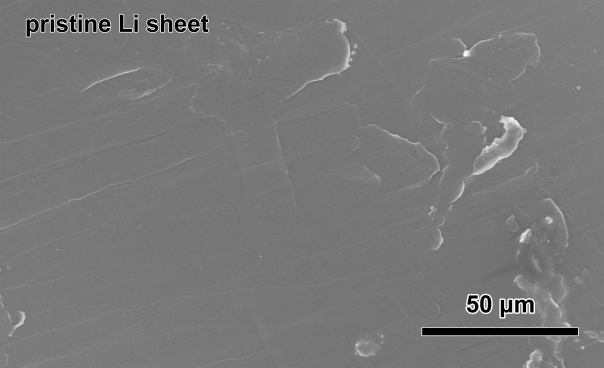


**Figure S20**. SEM image of the pristine Li sheet.


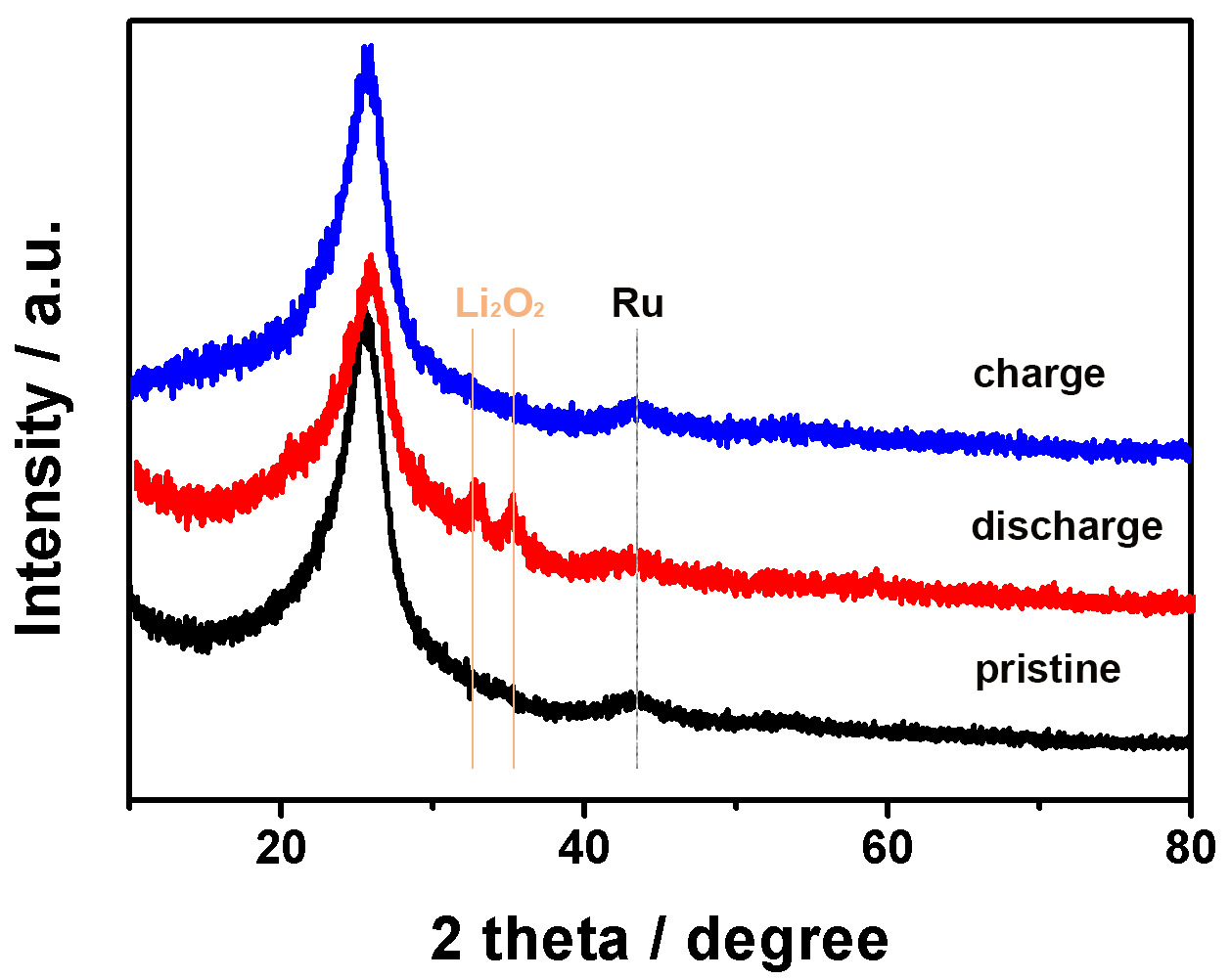


**Figure S21.** XRD patterns of cathodes at pristine, discharge, charge states.


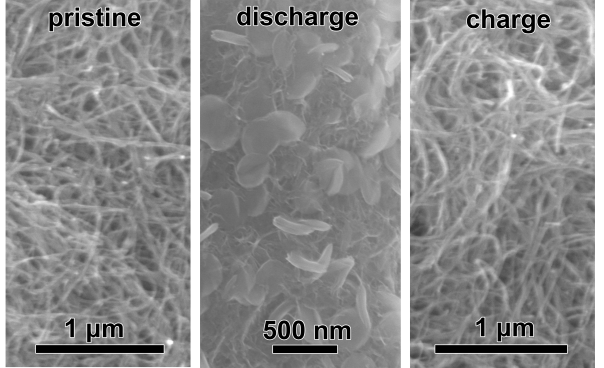


**Figure S22.** SEM images of cathodes at pristine, discharge, charge states.





**Figure S23.** SEM image of the HSE after the 40th cycle.


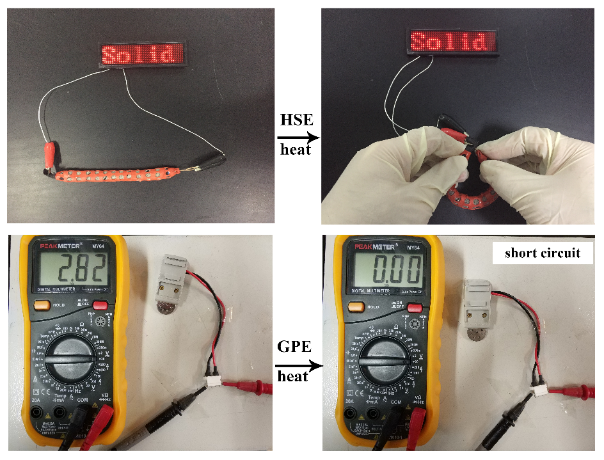


**Figure S24**. The safety experiment test for quasi-solid-state Li-O_2_ batteries with HSE and GPE electrolytes at 80 ^o^C for 10 min. Instead of cable-type battery for HSE, coin battery with GPE was used in consideration of safety.

**REFERENCES**

1. Li NW, Yin YX, and Yang CP *et al.* An artificial solid electrolyte interphase layer for stable lithium metal anodes. *Adv Mater* 2016; **28**: 1853-58.

2. Zhai H, Xu P, and Ning M *et al.* A flexible solid composite electrolyte with vertically aligned and connected ion-conducting nanoparticles for lithium batteries. *Nano Lett* 2017; **17**: 3182-87.

3. Zhang X, Liu T, and Zhang SF *et al.* Synergistic coupling between Li_6.75_La_3_Zr_1.75_Ta_0.25_O_12_ and poly(vinylidene fluoride) induces high ionic conductivity, mechanical strength, and thermal stability of solid composite electrolytes. *J Am Chem Soc* 2017; **139**: 13779-85.

4. Xu R, Zhang XQ, and Cheng XB *et al.* Artificial soft-rigid protective layer for dendrite‐free lithium metal anode. *Adv Funct Mater* 2018; **28**: 1705838.

5. Zhang W, Nie J, and Li F *et al.* A durable and safe solid-state lithium battery with a hybrid electrolyte membrane. *Nano Energy* 2018; **45**: 413-19.

6. Zhang ZY, Antonio RG, and Choy KL*.* Boron nitride enhanced polymer/salt hybrid electrolytes for all-solid-state lithium ion batteries. *J Power Sources* 2019; **435**: 226736.

7. Dixit MB, Zaman W, and Hortance N *et al.* Nanoscale mapping of extrinsic interfaces in hybrid solid electrolytes. *Joule* 2020; **4**: 207-21.

8. Liu XJ, Liu J, and Qian T *et al.* Novel organophosphate-derived dual-layered interface enabling air-stable and dendrite-free lithium metal anode. *Adv Mater* 2020; **32**: 1902724.

9. Lee DJ, Lee HK, and Kim YJ *et al.* Sustainable redox mediation for lithium-oxygen batteries by a composite protective layer on the lithium-metal anode. *Adv Mater* 2016; **28**: 857-63.

10. Yi J and Zhou HS. A unique hybrid quasi-solid-state electrolyte for Li-O_2_ batteries with improved cycle life and safety. *ChemSusChem* 2016; **9**: 2391-96.

11. Cho SM, Shim J and Cho SH *et al*. Quasi-solid-state rechargeable Li-O_2_ batteries with high safety and long cycle life at room temperature. *ACS Appl Mater Interfaces* 2018; **10**: 15634-41.

12. Liu SM, Zhang WQ and Chen N *et al*. Porous urchin-like Co_3_O_4_ microspheres as an efficient bifunctional catalyst for nonaqueous and solid-state Li-O_2_ batteries. *ChemElectroChem* 2018; **5**: 2181-5.

13. Kwak WJ, Park J and Nguyen TT *et al*. Dendrite- and oxygen-proof protective layer for lithium metal in lithium-oxygen batteries. *J Mater Chem A* 2019; **7**: 3857-62.

14. Liu T, Feng XL and Jin X *et al*. Hold up an “umbrella” for lithium metal anode towards a safe flexible lithium-air battery in ambient air. *Angew Chem Int Ed* 2019; **131**: 18408-13.

15. Shu CZ, Long JP and Dou SX *et al*. Component-interaction reinforced quasi-solid electrolyte with multifunctionality for flexible Li-O_2_ superior safety under extreme conditions. *Small* 2019; **15**: 1804701.
